# Rapid and Simple Detection of *Escherichia coli* by Loop-Mediated Isothermal Amplification Assay in Urine Specimens

**Published:** 2018

**Authors:** Reihaneh Ramezani, Zahra Kardoost Parizi, Nassim Ghorbanmehr, Hamideh Mirshafiee

**Affiliations:** 1. Department of Biomedical Sciences, Women Research Center, Alzahra University, Tehran, Iran; 2. Department of Biotechnology, Faculty of Biological Sciences, Alzahra University, Tehran, Iran; 3. Department of Microbiology, Faculty of Biological Sciences, Shahid Beheshti University, Tehran, Iran

**Keywords:** *Escherichia coli*, Isothermal amplification, LAMP, Urinary tract infection

## Abstract

**Background:**

To improve urinary tract infection detection, we evaluated the specificity and sensitivity of Loop-mediated isothermal Amplification Method (LAMP) for detection of the *Eschericia coli* *(E. coli)* in urine samples, for the first time.

**Methods:**

Primers were designed to target the malB gene of *Escherichia coli*. LAMP assay was performed on urine specimens collected from patients with urinary tract infection symptoms.

**Results:**

As expected, LAMP was more specific and sensitive than direct microscopic tests. LAMP assay showed the best detection limit of DNA copies with 1.02 copies.

**Conclusion:**

LAMP method offers several advantages in terms of sensitivity, rapidness and simplicity for detection of *E. coli* infection in urine samples. The LAMP method would be highly suitable for the early detection of the UTIs and also comfort quick diagnosis of UTI in clinical laboratories with limited equipment.

## Introduction

Diagnosis and early detection of Urinary Tract Infection (UTI) are critical factors in disease management. Urine culture is the gold standard for the detection of UTI, but this method is time consuming. Molecular techniques are the more sensitive and rapid diagnostic tools for detecting pathogens in clinical samples. Conventional amplification methods such as PCR, require sufficient skill and expensive devices. In this regard, several isothermal amplification methods for the rapid detection of UTIs have been developed such as Loop mediated isothermal Amplification method (LAMP) 1, Nucleic Acid Sequence Based Amplification (NASBA) 2, Self-sustained sequence replication (3SR), *etc*. Compared to Conventional amplification methods, isothermal amplification techniques especially LAMP is rapid and easy to use for early detection of DNA. The thermocyclers are not needed in this amplification method, so procedure can be completed under isothermal conditions 1,2.

In this study, the performance of LAMP based on the *mal B* gene was assessed to detect the presence of *Escherichia coli (E. coli)* in urine specimens.

## Materials and Methods

### Samples collection and bacterial culture

Urine specimens collected from patients referring to Resalat Hospital in Iran. *E. coli* (ATCC 25922) was used to evaluate the specificity and sensitivity of the LAMP reaction and grown in LB broth medium.

### DNA extraction

1.5 *ml* of urine was poured into a microfuge tube. The tubes were centrifuged at 14000 *rpm* for 10 *min*. After washing with PBS buffer, samples were centrifuged again. Remaining Precipitate was solved in PBS buffer and incubated at 90*°C* for 10 *min* then was subjected to reaction components of LAMP.

### LAMP primers

*E. coli*-specific Primers used for this study targeted malB gene with GenBank accession no. CP016358 which encodes Maltoprotein. A set of four specific primers (F3, B3 for outer primers and FIP, BIP for inner primers), as described by Hill *et al* 3, were employed for LAMP assay (Table 1).

**Table 1.** Primers used in this study

**Assay**	**Primer**	**Sequence (5′- 3′)**
**LAMP**	F3	5′-GCCATCTCCTGATGACGC-3′
	B3	5′-ATTTACCGCAGCCAGACG-3′
	FIP	5′-CTGGGGCGAGGTCGTGGTAT-TCCGACAAACACCACGAATT-3′
	BIP	5′-CATTTTGCAGCTGTACGCTCGC-AGCCCATCATGAATGTTGCT-3′

### LAMP reaction

Final concentration of the LAMP reaction mix was prepared in total 25 *μl* volume containing 0.2 *Mm* F3 and B3 primers, 1.6 *μM* FIP and BIP primers, 1.4 *mM* dNTPs mixture, 1 *mM* betaine (Sigma, Shanghai, China), 6 *mM* MgSO4, 1× LAMP buffer (New England Biolabs, Ipswich, MA), 320 *U/ml* Bst DNA polymerase (New England Biolabs). The reaction mixture was incubated at 66*°C* for 1 *hr* using heating block (HB-R48, Wisdcopany, Korea).

### Detection of amplification products

**Figure 1.**
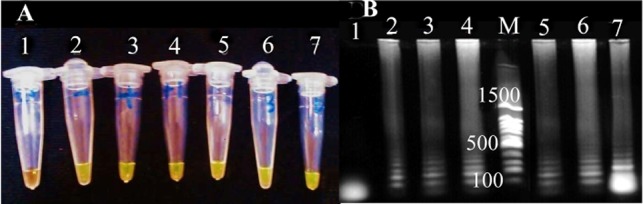
The detemination of LAMP products. A) visual detection after addition of SYBR green dye without UV illumination. Tube 1: negatine control, tube 2: positive control, tube 3, 4, 5, 6 and 7: LAMP amplification products from positive urine samples. B) Analysis on 1% agarose gel electrophoresis. lane 1: negatine control, lane 2: positive control, lane 3, 4, 5, 6 and 7: LAMP amplification products from positive urine samples., M: 100 *bp* DNA ladder.

To detect LAMP products, electrophoresis was performed in 1% agarose gel (Figure 1). The LAMP reaction evaluation was also done by addition Syber green (1:10 dilution of a 10,000× stock solution) to each reaction tubes and color changes was visualized by UV lamp (302 *nm*). Green color indicates a positive result (Figure 1).

### Specificity and sensitivity of LAMP

To determine the sensitivity of the assays, 10-fold serial dilutions purified DNA derived from *E. coli* ATCC 25922 (Table 2) were prepared. Extracted DNAs of several bacterial strains were examined to evaluate the specificity of the LAMP assay.

**Table 2.** Concentration and number of DNA copy for each sample

**Samples**	**Dilution**	**Number of DNA copy**
**A**	10^−1^	1.02×10^7^
**B**	10^−2^	1.02×10^6^
**C**	10^−3^	1.02×10^5^
**D**	10^−4^	1.02×10^4^
**E**	10^−5^	1.02×10^3^
**F**	10^−6^	1.02×10^2^
**G**	10^−7^	1.02×10^1^
**H**	10^−8^	1.02

### Microbiological and molecular Examinations of the urine samples

Macroscopic urinalysis was done by direct visual examination of the urine samples to which a small amount of acid was added. For microbiological tests (Table 3), all urine samples collected from patients were grown in LB broth medium. To determine whether there are *E. coli*, all of the urine samples were cultured on Eosin Methylene Blue Agar (Merck, Germany). The observation of green metallic sheen was considered positive. For LAMP assay (Table 3), 1 *ml* of each urine samples was centrifuged at 15000 *rpm*, 4*°C* for 10 *min*. Then, the resultant precipitate was resuspended in sterile water. Once incubated at 90*°C* for 10 *min*, the suspensions were added to LAMP reactions.

**Table 3.** The results of all experiments (turbidity, culture, PCR and LAMP) for clinical specimens

**Samples**	**Sex**	**Age**	**Turbidity**	**Culture**	**LAMP result^a^**
**C1**	F	35	+	+	+
**C2**	F	45	+	+	+
**C3**	F	63	+	−	−
**C4**	F	12	+	−	−
**C5**	M	36	+	−	−
**C6**	F	42	+	+	+
**C7**	M	43	+	−	−
**C8**	F	56	+	−	−
**C9**	F	37	+	+	+
**C10**	F	59	−	−	−
**C11**	F	63	+	+	+
**C12**	M	60	+	−	+
**C13**	F	57	+	+	+
**C14**	M	33	+	+	+
**C15**	F	40	+	+	+
**C16**	F	64	+	+	+
**C17**	M	39	+	+	+
**C18**	M	66	+	+	+
**C19**	F	27	+	−	−
**C20**	M	35	+	+	+

a +, amplification occurred; −, amplification did not occur.

## Results

**Figure 2.**
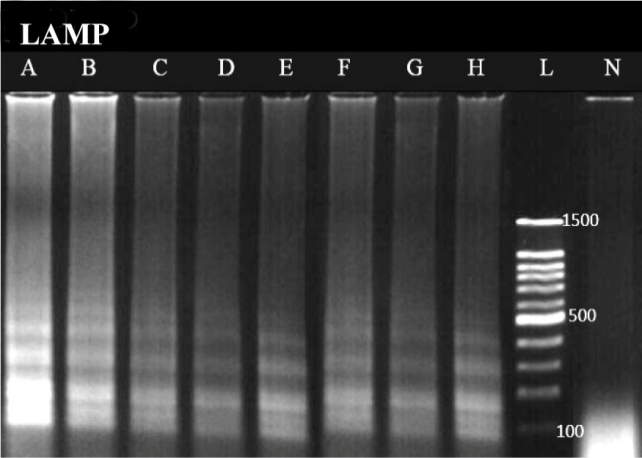
Sensitivity analysis of LAMP based on number of DNA copy, The lanes labeled as A, B, C, D, E, F and G, correspond to the table 2, A: 10^−1^ dilution, B: 10^−2^, C: 10^−3^, D: 10^−4^, E: 10^−5^, F: 10^−6^, G and H: 10^−7^ (The DNA copy number of the first dilution is 1.02×10^7^). L: 100 *bp* DNA Ladder. N: negative control.

LAMP assay showed the best detection limit of DNA copies with 1.02 copies (10^−8^ dilution) (Figure 2). In this study, DNA copy number of each dilution was calculated using the following formula 4:

$$\text{DNA}\;\text{copy}\;\text{number}=\frac{\left(6.022\times 10^{23})\times C(\mathrm{ng}\right)}{650(1\times 10^{9})\times \mathrm{DNA}\;\mathrm{length}}$$

**Figure 3.**
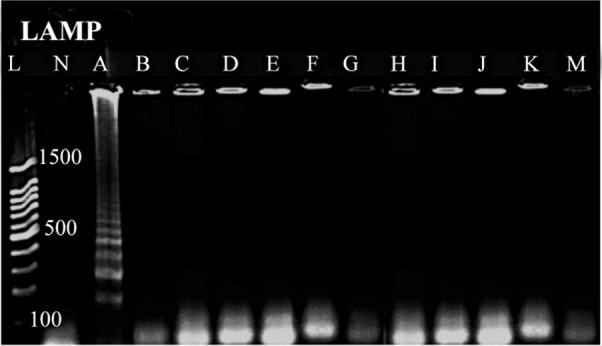
Specificity analysis based on genus-specific primer for *E. coli*, A) *Escherichia coli,* B) *Shigella spp*., C) *Acinetobacter spp.,* D) *Staphylococcus aureus,* E) *Proteus mirabilis,* F) *Entrococcus faecalis*, G) S*treptococcus epidermidis*, H) *Streptococcus pyogenes*, I) *Kelebsiella pneumonia,* J: *Pseudumonas p2,* K: *Salmonella typhimurium*, M: S*taphylococcus saprophiticus*. L) 100 *bp* DNA Ladder, N) negative control.

In which C is concentration of the extracted DNA. DNA length is bases pair number of the selected bacterium which was 5130767 base pairs for *E. coli* ATCC 25922 in our study. No cross reaction was seen with other bacteria, indicating that designed primers of LAMP assay were specific for *mal B* gene (Figure 3).

As expected, LAMP was more specific and sensitive than direct microscopic tests. Among the 20 urine samples, 12 cases were positive on EMB agar, but 13 cases were positive by LAMP. We were not capable of detecting *E. coli* contaminated urine sample by culturing. The reason of this observation could be inability of bacteria to grow on culture medium due to antibiotics consumption by patients. Since DNA is stable for long time and it is detectable after bacteria death, so it would be expectable that DNA-based molecular detection techniques like LAMP could detect DNA in antibiotic- killed bacteria as well.

## Discussion

Diagnosis of UTI is often carried out by clinical symptoms, microscopic and culture examinations. Direct microscopic diagnosis has a high error rate (approximately 31%) and culture based methods are time consuming 5. The advantages of nucleic acid amplification based methods such as PCR, are their speed and sensitivity in pathogen detection, but these assays need trained users and some expensive devices 6. In this study, one of isothermal nucleic acid amplification methods called LAMP was employed to overcome the mentioned problems in UTIs diagnosis procedures.

LAMP could recognize target gene by 4–6 primers and the amplification can be done in constant temperature for 60 *min*. Unlike with PCR, there is no need for thermocycler and heat denaturation of the double strand DNA 7.

There are some similar researches in which LAMP assay has been targeted different genes to detect various strains of *E. coli* such as the *stx1, stx2* 8, *aggR* 9, *ipaH* 10, the *heat-labile I* *(LTI)* and *heat-stable I* *(STI)* genes 11 and *mal B* 3. Although many researches have been done to detect *E. coli* by LAMP method, but there is not any report for employing this technique to identify *E. coli* in urine samples. In spite of more than 90% similarity between *Shigella* and *E. coli*, no cross reaction was observed between them in this study. The reason of high specificity of LAMP method is recognition of six distinct sequences on the target DNA by a set of four specially designed primers 1.

## Conclusion

In conclusion, the present study suggests that LAMP method would be highly suitable for the detection of the UTIs and also comfort quick diagnosis of UTI in clinical laboratories with limited equipment. However, LAMP assay is preferable to microbiological culture in terms of speed, simplicity and sensitivity.
